# Baraneck's Tuberculin

**Published:** 1907-08-10

**Authors:** 


### BARANECK'S TUBERCULIN.

For the last ten years Professor Baraneck has been investigating the properties of his tuberculin, discovered in 1894, and his work deserves the attention of the profession generally, not only because of the results which he has obtained but equally because of the self-sacrificing zeal which he has displayed in his investigations. Ocupying the chair of Zoology in the Academy of Natural Sciences at Neuchatel, Professor Baraneck has pursued his labours during these ten years with a quiet, unostentatious perseverance that is the hall-mark of sincerity in original research.

The laborious elaboration of a tuberculin of which the curative value was exceedingly problematical —in view of the non-success of Koch and others— was not an undertaking which most scientists would have embarked upon unaided by State or other financial support. Baraneck, however, kept his object steadily in view, and spared neither time nor expense in investigating the properties of his antitoxin, and now, after ten years of experiment and research, he has laid the results of his investigations before the profession, and, with his helpers, presented to the Swiss Medical Congress, recently in session at Neuchatel, a *résumé* of his conclusions.

Baraneck's tuberculin is not, strictly speaking, an anti-toxin. It is in no way analogous to Behring's anti-diphtheritic serum. It is a mixture, rather, of two active toxins, the one derived from the bacilli themselves and the other elaborated in the culture medium in which they were grown. The object is to excite an active infective process in the tissues of the organism into which it is injected. This infective process gives rise to a reaction of the organism itself, which to protect itself manufactures an anti-toxin, and so gradually acquires a resisting power against tubercular infection. For some time past, Professor Baraneck, not content with his own observations, has supplied samples of his tuberculin to various colleagues, and their reports are encouraging, though much more must be done before we are in a position to pass judgment upon the new remedy. Sahli of Berne, who has used the tuberculin supplied by Baraneck for several years, is convinced of its value "within certain limits and provided that proper precautions are taken in its administration." He insists, for instance, on the necessity for scrupulous aseptic precautions in making the injections, and upon the use of extremely attenuated initial doses. Coulon, again, who has used the tuberculin extensively in treating tuberculous patients at Neuchatel, reports encouraging results following the injection of graduated doses in cases of tuberculous bone lesions and general surgical tuberculosis. He makes his injection directly into the lesion, where it "almost immediately gives rise to an inflammatory reaction, leading to suppuration and final resolution." He has obtained striking results in the treatment of tubercular joint lesions, and at the Congress he exhibited cases of Pott's disease, tuberculous disease of the knee, ankle, wrist, and testicle treated in this manner with fairly satisfactory results. The treatment extends over a few weeks, the patient receiving, on an average, two injections per week, and being allowed to go about, and if possible to do his ordinary work.

Hitherto Baraneck's tuberculin appears to have been used only in cases of surgical tuberculosis, and there is no record of any experience of its use in pulmonary or visceral tuberculosis. The results obtained by its means in skeletal tuberculous lesions, however, are so encouraging that it seems worth while trying its effect upon cases of pulmonary tuberculosis or tubercular peritonitis. The results of these investigations, which are now being made at Neuchatel, will be awaited with interest by the profession, and if they are as valuable as those hitherto obtained, Baraneck's tuberculin will certainly be worthy the attention of the general practitioner.

